# Partial sequence of L1 gene in HPV 16 and HPV 18 isolated from head-neck squamous cell carcinoma tissues in Bangladesh

**DOI:** 10.1128/mra.00298-26

**Published:** 2026-09-14

**Authors:** S. M. Rashed Ul Islam, Md. Sayeam Khandaker, Bishnu Pada Dey, Syed Farhan Ali Razib, Kabir Hossen, Joy Prokash Debnath, Shamim Ahmed, Afzalun Nessa

**Affiliations:** 1 Department of Virology, Bangladesh Medical University 74464, Dhaka, Bangladesh; 2 Department of Biochemistry and Molecular Biology, Shahjalal University of Science and Technology 113074, Sylhet, Bangladesh; 3 Department of Pathology, Bangladesh Medical University 74464, Dhaka, Bangladesh; 4 Department of Otolaryngology-Head and Neck Surgery, Bangladesh Medical University 74464, Dhaka, Bangladesh; Queens College Department of Biology, Queens, New York, USA

**Keywords:** human papillomavirus, HPV-16, HPV-18, HNSCC, L1 gene, partial gene, Bangladesh

## Abstract

Human papillomavirus is one of the most crucial determinants responsible for the progression of head-neck squamous cell carcinoma (HNSCC). Here, we report partial L1 gene sequences of two high-risk etiological agents, HPV-16 and HPV-18, extracted through biopsies from multiple head-neck tissue regions of HNSCC patients in Bangladesh.

## ANNOUNCEMENT

Human papillomaviruses (HPVs), taxonomically, are members of the “Papillomaviridae” family (1), which structurally contain small, double-stranded monopartite circular gDNA containing the early, long control region, and late regions (2). Within the α-papillomavirus genus, HPV-16 and HPV-18 are the principal mucosal oncogenic viruses, also categorized as high-risk strains, that trigger head-neck squamous cell carcinoma (HNSCC) worldwide (3, 4). Globally, more than 30% of HNSCC cases occur due to HPV invasion (5), where the ratio is increasing steadily in the Asian subcontinent (6). To date, there are two published studies on HPV-associated HNSCC in Bangladesh. Notably, no publicly available sequence data of HPV extracted from HNSCC samples in Bangladesh have been reported, thereby limiting comparative genomic analyses in this region (5, 7). In this study, the partial sequence of the L1 gene of HPV-16 and HPV-18 isolated from head-neck tissue was determined through Sanger sequencing.

Initially, a total of 417 formalin-fixed paraffin-embedded (FFPE) tissues and fresh biopsy tissues were collected from several head-neck regions, where 214 specimens were histopathologically identified as HNSCC-confirmed cases. The HPV DNA was extracted from these samples using QIAamp DNA FFPE Tissue Kit (8) for FFPE tissues and QIAamp DNA Mini Kit (9) for fresh tissues. Consequently, the presence of HPV in HNSCC tissues was determined via the NoeDx HPV 26 Screening PCR Kit (10), and genotyping was also conducted using the NoeDx HPV 14 Genotyping Kit (11). Nested PCR was performed using MY09/MY11 and GP5+/GP6+ primer sets (12) under the optimal conditions (13), yielding a 140 bp amplicon. Subsequently, Sanger sequencing was conducted targeting the conserved HPV L1 gene.

Amplified PCR products with prominent DNA bands in gel electrophoresis were purified through ExoSAP-IT Express PCR product purification kit (14). Afterward, the purified samples underwent cycle sequencing utilizing BigDye Terminator v3.1 Cycle Sequencing Kit. Cycle sequencing products were further purified via a magnetic bead-based purification method. Additional verification was performed using the pGEM-3Zf(+) plasmid as a sequencing control. Capillary electrophoresis was conducted using the automated ABI 3500xL genetic analyzer (15), and we used the BDx_Std_Seq_50_POP7_Z module for sequencing. The sequences were trimmed using BioEdit v7.7.1 to remove noise and poor-quality chromatogram regions, while ambiguous or discordant peaks were verified by aligning the reverse-strand chromatogram. Subsequently, consensus sequences were generated using Mega software (16). Sequences were accepted based on interpretable chromatograms, concordant forward/reverse reads, resolved ambiguous bases, and BLASTn confirmation of HPV-16/HPV-18 L1 sequence identity. HPV DNA was detected in 24.8% of the total HNSCC-confirmed samples, with HPV-16 being the most prevalent (94.3%), and HPV-18 present in a small number (13.2%) of samples. Among the total HPV-positive samples, 7.5% were co-infected with both strains. The L1 gene sequences from the first 12 samples (Table 1) showed 100% nucleotide identity to the reference HPV-16 sequences, which were previously reported from Asian-American and African lineages in the NCBI database. Exclusively, PGV-24-116 showed 100% nucleotide identity to the HPV-18 reference sequence (Table 1).

**TABLE 1:** List of HPV16 and HPV 18 isolate sequenced data released to NCBI GenBank

SL no	Isolate ID	Accession no	HPV variant	Isolation source	Reference gene
01	PGV-24-106	PV107410.1	HPV 16	Buccal mucosa	MG850084.1
02	VRL-24-71	PV113111.1	HPV 16	Buccal mucosa	HE798674.1
03	VRL-24-95	PV113112.1	HPV 16	Buccal mucosa	MG850688.1
04	VRL-24-105	PV113113.1	HPV 16	Tongue	KY502130.1
05	VRL-24-121	PV113114.1	HPV 16	Tongue	MG849220.1
06	VRL-24-133	PV113115.1	HPV 16	Buccal mucosa	MG849811.1
07	VRL-24-157	PV126527.1	HPV 16	Buccal mucosa	MG849811.1
08	VRL-24-167	PV126528.1	HPV 16	Buccal mucosa	MG849811.1
09	VRL-24-180	PV126529.1	HPV 16	Buccal mucosa	KX947283.1
10	VRL-24-91	PV126530.1	HPV 16	Buccal mucosa	JN617890.1
11	VRL-24-123	PV126531.1	HPV 16	Tongue	JN617890.1
12	VRL-24-181	PV126532.1	HPV 16	Buccal mucosa	JN617901.1
13	PGV-24-116	PV357593.1	HPV 18	Buccal mucosa	AF548838.1

This study suggests that the rate of HPV-associated HNSCC in Bangladesh is apparently increasing compared to the previous studies (5, 7). As Bangladesh has a paucity of studies on HPV-associated HNSCC with no recent investigation, this study could lay the groundwork for reinitiating future research in this area by the scientific and medical community.

## Data Availability

The partial consensus sequences generated from forward and reverse Sanger reads have been submitted to GenBank of the National Center for Biotechnology Information (NCBI) under the following accession numbers: PV107410.1, PV113111.1, PV113112.1, PV113113.1, PV113114.1, PV113115.1, PV126527.1, PV126528.1, PV126529.1, PV126530.1, PV126531.1, PV126532.1, and PV357593.1, and the raw reads have been deposited in the Sequence Read Archive under BioProject PRJNA1464148.
